# The Select Committee on Secret Remedies

**Published:** 1912-04-20

**Authors:** 


### The Hospital

The Workers' Newspaper of Administrative Medicine and Institutional Life, Administration, National Insurance and Health.

No. 1345, Vol. LII. SATURDAY, APRIL 20, 1912. PRICE ONE PENNY.

### THE SELECT COMMITTEE ON SECRET REMEDIES.

The official investigation of the traffic in this country in patent medicines, which has been long overdue, is to be commenced at once, and no time must be lost by those who are going to present the case against secret remedies to the Select Committee, nominated by the Home Secretary, in completing their preparations. The conflict will be between personal interests and the public health; on the one side will be arranged all the skill which the profits on patent medicines can pay for, and on the other a small body of reformers whose simple desire it is to put an end to a peculiarly cruel fraud. The owners of proprietary medicines, who are fighting for a retention of privileges which they have grossly abused, have much to lose; their opponents are striving to establish a principle, but otherwise have nothing to gain. For years the makers of advertised medicines have been preparing their defence, foreseeing that the attack could not be delayed much longer. Unfortunately they have been furnished with one of their arguments by those members of the medical profession who have not been guiltless of prescribing proprietary products of secret composition; for so long as quackery is condoned by regular practitioners, laymen can hardly be expected to condemn it.

It was stated in one of a series of articles which appeared in The Hospital four years ago that of five hundred prescriptions dispensed in London chemists' shops a hundred and five were wholly or partly for branded products, a large proportion of which were compounds whose composition is known only to the makers. That is a fact which may be capable of explanation, and the medical opponents of quackery must be prepared to explain it, for the quacks are sure to use it in support of their case. In hospital practice where young practitioners make their earliest essays at prescribing, the use of proprietary products is discouraged, but from the moment they begin to practise privately every effort is made to induce them to forsake the Pharmacopœia. There is, of course, no comparison between nostrums publicly advertised to cure all sorts of diseases and the majority of the proprietary compounds which practitioners are persuaded to prescribe. Some of the latter are extremely valuable additions to materia medica, and no objectionable methods are used in bringing them to the notice of practitioners. The Committee should find little difficulty in distinguishing between the various classes of products. The manufacturer who discovers some new process which is, from the medical standpoint, of real value has as much right as any other inventor to the protection afforded by the Patents Act. He discloses his process, and on taking out Letters Patent is granted an absolute monopoly in the working of his invention for a term of fourteen years. His conduct is not improper and the taint of secrecy does not attach to his product. On the other hand, the Committee should be asked to consider the question whether a manufacturer ought to have the unlimited protection afforded by trade marks; for the purpose of the trade-mark laws there need be no value in the product, and no disclosure of the nature of the article sold under the trade-mark need be made. The classes of products, however, prescribed by medical men are wholly different in character from those advertised in the public Press, and it is to the latter that the attention of the Select Committee will be mainly directed. The vastness of the traffic in these articles is remarkable: in England, Wales, and Scotland the public spends approximately two and a half million pounds sterling on their purchase every year, or considerably more than half the sum allocated by the Government actuaries to pay for the medical attendance on, and medicines for, fifteen million people insured under the National Insurance Act.

The traffic has grown to such enormous proportions because there has been nothing to impede its growth, for while in practically every other civilised country the sale of proprietary medicines is subject to restrictions, it is subject to no legal and practically no social restraint in Great Britain. The law concerning secret medicinal compounds on the Continent, in America, and our own Colonies was explained in the series of articles already referred to, and all that need be added to those statements is that the tendency during the last few years has been to enforce the law more rigorously than ever. To prevent the use of false trade descriptions proceedings could be taken under the Merchandise Marks Act, and there are many palpable cases of obtaining money by false pretences which could be dealt with in another way. Section 27 of the Sale of Food and Drugs Act, 1875, which provides that "every person who shall wilfully give a label with any article sold by him which shall falsely describe the article sold shall be guilty of an offence under this Act and be liable to a penalty not exceeding £20," could also be enforced if the local authorities showed any inclination to protect the public from this kind of fraud. The Royal College of Physicians urged Mr. Churchill, when he was Home Secretary, to consider the desirability of compelling manufacturers and vendors of "patent medicines" to print on the labels the exact composition of such medicines and the amount of each constituent, and of prohibiting them from printing the names of the diseases or symptoms which the medicines purport to cure. The College will presumably support this demand by evidence before the Committee; and while the regulations suggested are more drastic than those existing in other countries, it does not seem too much to ask if the traffic is to be kept within something like legitimate limits. Even in the event of the inquiry leading to no improvement, which is hardly credible, there is some reason to hope that the operation of the National Insurance scheme will have a very damaging effect on quackery. When fifteen millions of the quacks' best customers are in a position to obtain proper medical advice and properly dispensed medicines without extra payment, there will surely be a heavy decrease in the sale of nostrums.

